# Fabry Disease and the Effectiveness of Enzyme Replacement Therapy (ERT) in Left Ventricular Hypertrophy (LVH) Improvement: A Review and Meta-Analysis

**DOI:** 10.7150/ijms.66448

**Published:** 2022-01-01

**Authors:** Chung-Lin Lee, Shuan-Pei Lin, Dau-Ming Niu, Hsiang-Yu Lin

**Affiliations:** 1 Department of Pediatrics, MacKay Memorial Hospital, Taipei, Taiwan.; 2 Institute of Clinical Medicine, National Yang-Ming Chiao-Tung University, Taipei, Taiwan.; 3 Department of Medicine, MacKay Medical College, New Taipei City, Taiwan.; 4 MacKay Junior College of Medicine, Nursing and Management, Taipei, Taiwan.; 5 Department of Rare Disease Center, MacKay Memorial Hospital, Taipei, Taiwan.; 6 Division of Genetics and Metabolism, Department of Medical Research, MacKay Memorial Hospital, Taipei, Taiwan.; 7 Department of Infant and Child Care, National Taipei University of Nursing and Health Sciences, Taipei, Taiwan.; 8 Taiwan Clinical Trial Consortium in Fabry Disease.; 9 Department of Pediatrics, Taipei Veterans General Hospital, Taipei, Taiwan.; 10 Department of Medical Research, China Medical University Hospital, China Medical University, Taichung, Taiwan.

**Keywords:** Enzyme replacement therapy, Fabry disease, Left ventricular hypertrophy, Meta-analysis

## Abstract

**Background:** Fabry disease is an inherited lysosomal storage disease affecting multiple organs with complications, including cardiomyopathy such as left ventricular hypertrophy (LVH). Enzyme replacement therapy (ERT) has been the main treatment for Fabry patients since 2001. However, the indications of ERT are not clearly defined. We performed a meta-analysis according to previous studies to review the benefit of ERT for LVH improvement in Fabry patients.

**Methods:** We performed a literature search from the National Center for Biotechnology Information (NCBI) and PubMed database without restriction of years for systematic review purposes. We performed a systematic review of clinical cohort studies and trials using a pooled analysis of proportions. We calculated the pooled proportions and the confidence intervals (CI) for left ventricular mass index (LVMI) for both ERT treatment and ERT treatment-naïve groups. The results for before ERT treatment and after ERT treatment are also investigated.

**Results:** A total of 5 cohort studies and 2 randomized controlled trials (RCTs), involving a total of 552 participants (267 on ERT treatment versus 285 on naïve treatment), met the inclusion criteria. The pooled proportions analysis showed that the difference in means of LVMI between the ERT treatment group and the ERT treatment-naïve group was -0.149 [95% CI: -0.431, 0.132]. Effect differences favored the ERT treatment group over the ERT treatment-naïve group (*p* = 0.034). Another analysis included 3 cohort studies and 1 RCT with 442 participants (228 on before ERT and 214 on 4 years after ERT). The pooled proportions analysis showed that the difference in means of LVMI between the before ERT treatment group and the after ERT treatment group was -0.448 [95% CI: -0.787, -0.108]. It favored the 4 years after ERT group over the before ERT group (*p* = 0.037).

**Conclusions:** Based on the currently available data, our meta-analysis showed that there are beneficial effects on LVH improvement with ERT in Fabry disease patients. It is better to start ERT as soon as we have diagnoses in female carriers and atypically affected males. Further research is needed to investigate the role of ERT in LVH improvement.

## Introduction

Fabry disease (FD) is a rare genetic disease with an X-linked inherited pattern. It is caused by a deficiency of alpha-galactosidase A (ɑ-Gal A) due to mutations in the *GLA* gene. The underlying pathophysiological mechanism of FD is still incompletely understood. Nevertheless, the accumulation of globotriaosylceramide (Gb3) caused by the deficiency of ɑ-Gal A may cause adverse effects on the endothelial and smooth cells of the intima and the media of small arterioles. Other cells, including cardiomyocytes, valvular cells, tubular and glomerular cells, and nerve cells, would experience similar effects 1. Organ damage occurs when FD progresses, especially in the cardiovascular system, nervous system, and kidneys.

According to previous studies, about three to nine percent of male patients with unexplained hypertrophic cardiomyopathy have low serum levels of ɑ-Gal A. This indicates that these unexplained hypertrophic cardiomyopathy patients may have undiagnosed FD 2-4. In addition to left ventricular hypertrophy (LVH), there are other cardiovascular manifestations of FD, including symptomatic valvular abnormalities, arrhythmias, and conduction disturbances 5. Cardiac abnormalities may be the predominant manifestations in atypical type FD 6,7.

Enzyme replacement therapy (ERT) has become the main therapy of FD since 2001. It has effects on cardiac structure, including progressive decreases in the interventricular septum (IVS) thickening and in the left ventricular (LV) mass 8-11. However, there are not many systematic reviews focusing on the improvement of LVH after ERT. This is an important issue, as the regression of cardiac hypertrophy could reduce morbidity and mortality in FD patients. In this review, we focus on Fabry disease and LVH. We also perform a meta-analysis regarding the effectiveness of ERT in LVH improvement.

## Methods

**Figure 1:**
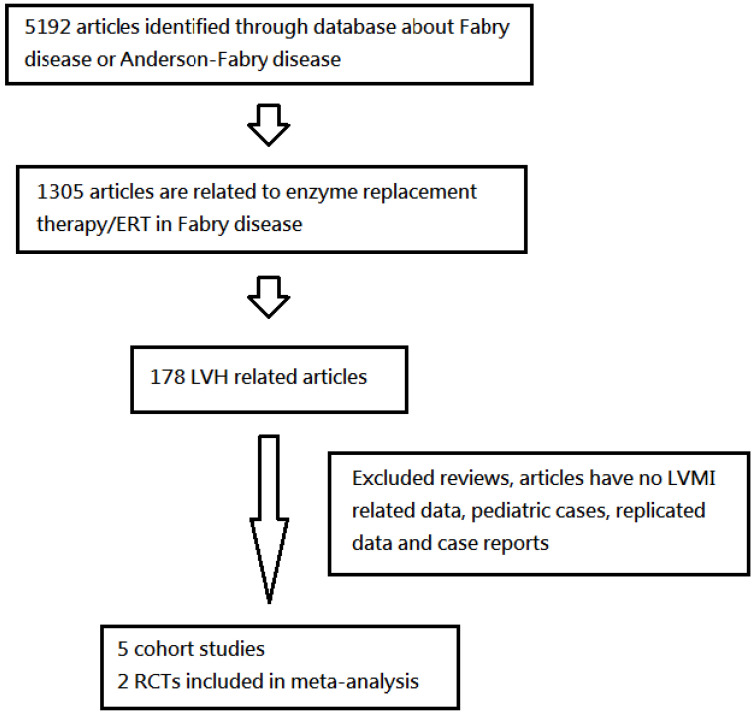
A flow-sheet of systematic review on left ventricular hypertrophy (LVH) from database search, including enzyme replacement therapy (ERT) group and naïve treatment group. Another analysis groups were 4 years after ERT group and before ERT group (LVMI: left ventricular mass index).

We referenced the National Center for Biotechnology Information (NCBI) and U.S. National Library of Medicine National Institutes of Health (PubMed) databases based on the terms “Fabry Disease,” “left ventricular hypertrophy,” “enzyme replacement therapy,” “cardiomyopathy,” “Agalsidase Alfa,” and “Agalsidase Beta.” We found research involving randomized controlled trials (RCTs) and primary studies without restriction of years. Moreover, we screened all titles and abstracts and obtained full text articles of all potentially associated studies or trials. Ultimately, we analyzed five clinical cohort studies and two RCTs via a pooled analysis of proportions (Figure 1).

### Eligibility criteria

Study design: Studies were classified as cohort studies or RCTs. We excluded observation studies, case series, case reports, and results from other systematic reviews.

Study group: FD patients were identified via genetic analysis, enzyme analysis, and/or biopsy. The genotypes included IVS4+919G>A, W204X, E398DfsX6, G132R, S345X and IVS4+919G/A. They either underwent ERT treatment (either Agalsidase Alpha or Agalsidase Beta) or were ERT treatment naïve. We excluded studies on children and studies including duplicate data.

Primary outcomes: FD patients had LVH, which was predefined as a left ventricular mass index (LVMI) higher than the upper normal limit (men, ≥115 g/m^2^; women, ≥95 g/m^2^) 12, with or without ERT. Another analysis groups were patients before and 4 years after ERT. We excluded studies which did not report LVMI outcome data.

### Proportional meta-analysis

We performed a meta-analysis to analyze all these studies for the results of LVMI in FD patients with or without ERT (5 clinical cohort studies and two RCTs) and before ERT or 4 years after ERT (3 clinical cohort studies and one RCT). A random-effects model was developed and applied to the two groups of ERT treatment and ERT treatment-naïve. Another two groups were the patients before ERT and 4 years after ERT. This meta-analysis was performed in the Comprehensive Meta-Analysis software package (Version 3).

## Results

Table 1 showed the numbers of patients, mean ages, mean follow-up years and genders encompassed by this meta-analysis. All patients were in their 30s and 40s, and their mean age was 43.4 years. Among the seven studies selected, four studies specified patient phenotypes as classic, and the others did not. There were four studies conducted in Europe, one in North America, and two in Asia.

**Figure 2:**
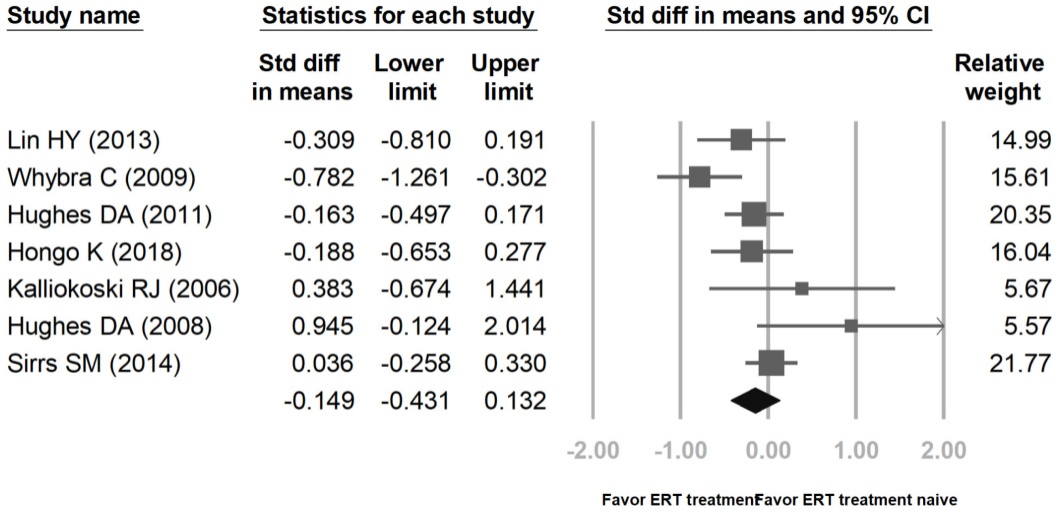
Proportional meta-analysis result for a pooled proportion from 5 cohort studies and 2 randomized controlled trials (RCTs) for left ventricular mass index (​LVMI) in Fabry disease. The pooled proportions analysis showed that the difference in means of LVMI between the enzyme replacement therapy (ERT) group and the ERT treatment-naïve group was -0.149 [95% CI; -0.431, 0.132; I^2^ = 55.99%;* p* = 0.034].

**Figure 3:**
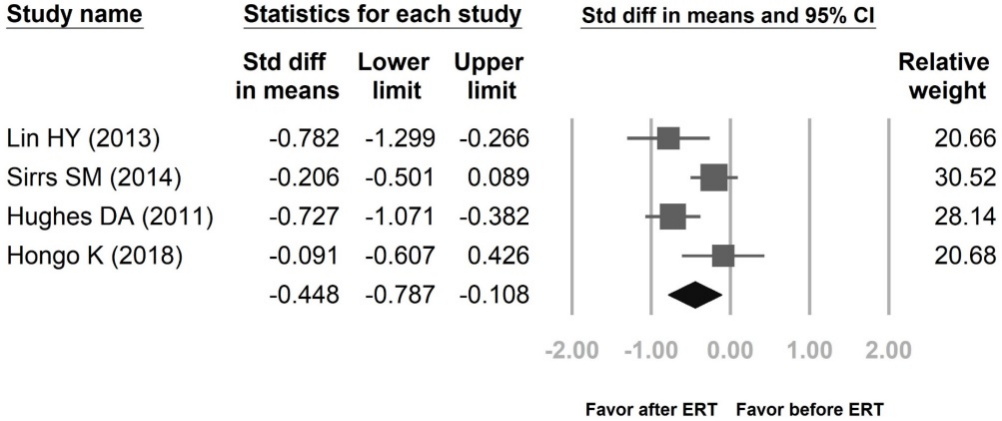
Proportional meta-analysis result for a pooled proportion from 3 cohort studies and one randomized controlled trial (RCT) for left ventricular mass index (​LVMI) in Fabry disease. The pooled proportions analysis showed that the difference in means of LVMI between the 4 years after enzyme replacement therapy (ERT) group and the before ERT group was -0.448 [95% CI; -0.787, -0.108; I^2^ = 64.75%;* p* = 0.037].

Figure 2 showed proportional meta-analysis result for a pooled proportion from 5 cohort studies and 2 RCTs for LVMI in Fabry disease. The inconsistency level (I^2^) of the random-effects model is 55.99% with high heterogeneity, showing that the variation within both groups is due to heterogeneity rather than random chance. Thus, it is better to use a random-effects model than a fixed-effects model in this study. Figure 3 revealed proportional meta-analysis result for a pooled proportion from 3 cohort studies and one RCT for LVMI in Fabry disease. The inconsistency level (I^2^) of the random-effects model is 64.75% with high heterogeneity. As Figure 2, it is better to use a random-effects model than a fixed-effects model.

The pooled proportions for LVMI were based upon ERT treatment and ERT treatment-naïve groups from five cohort studies 13-17 and two RCTs 18,19, including a total of 552 patients (ERT treatment: 267, ERT treatment-naïve: 285). The pooled proportions analysis showed that the difference in means of LVMI between the ERT treatment group and the ERT treatment-naïve group was -0.149 [95% CI: -0.431, 0.132]. Effect differences favored the ERT treatment group over the ERT treatment-naïve group (*p*= 0.034). Another analysis groups were patients before ERT and 4 years after ERT. These results were according to three cohort studies 13,15-16 and one RCT 19, including a total of 442 patients (before ERT: 228, 4 years after ERT: 214). The difference in means of LVMI between the before ERT group and the 4 years after ERT group was -0.448 [95% CI: -0.787, -0.108]. Effect differences favored the 4 years after ERT group over the before ERT group (*p*= 0.037).

## Discussion

LVH is one of the leading causes of the morbidity and mortality associated with FD patients 20. The accumulation of Gb3 within myocytes, the coronary endothelium, intimal and medial cells, and valve tissue is the main factor relating to the pathological changes observed in the hearts of FD patients. This accumulation causes LVH, conduction abnormalities, valve thickening, heart failure, anginal chest pain, and (less frequently) acute myocardial infarctions. LVH is also an independent risk factor for cardiovascular events and premature mortality 21. Therefore, the significant reduction of LVMI after 6 months of ERT is clinically relevant. ERT had a positive therapeutic effect (regression or stabilization of LV hypertrophy) in FD patients. However, due to the higher variability of measurements by echocardiography, the change in LVMI lacks statistical significance.

Some studies showed that ERT could result in a significant regression in the LVH of FD patients in a short time 22,23; however, some studies suggested only a slight improvement in the LVH after ERT 24-26. In some reports, there was not any initial improvement within a few years after the initiation of ERT 22-24. On the contrary, we observed a gradual increase in the LVMI after long-term ERT 17-19. We do not know what reason caused the different changes in the LVMI after ERT. However, Gb3 decreased in the cardiac myocytes over a period of a few years after ERT 27. A previous study showed that the prevalence of LVH in male patients was higher than that in female patients 28. It was caused by that female FD patients had a wide range of a-Gal A activity overlapping that in normal healthy people. Further evidence indicated that ERT might improve the degree of LVH. Ortiz et al. 29 suggested that after starting ERT, there was a “lag time” before a clinical benefit was realized. They followed 1,044 FD patients for 5 years, in which the incidence of severe clinical events was 111/1000 patient-years in the first 6 months. Then, the incidence decreased and remained stable in the range of 40-58/1000 patient-years after 6 months (*p* < 0.05). However, this study had a limitation in that it combined all severe clinical events together, rather than separating out LVH. There was also a pooled analysis conducted in 2017 that favored ERT treatment 30. This study showed that the cardiovascular complication rate of Agalsidase Beta was lower in treated patients than in untreated patients (7.0% versus 26.2%; *p* < 0.05). Nevertheless, a limitation of this study was that there was significant heterogeneity in the clinical outcomes of all studied groups. There was also selection bias in the data, as there were no RCTs included 30.

We analyzed RCTs and well-designed cohort studies to decrease the heterogeneity in our meta-analysis. According to a previous study, there was no significant difference between the groups regarding clinical events in two trials 31. Due to this reason, we classified ERT treatment as one group instead of two separate treatment groups to increase the power of our meta-analysis. Our pooled analysis showed that ERT has a beneficial effect on the improvement of LVMI in FD patients. However, due to the lag time of ERT, the effects of ERT shows slowly. FD patients with LVH but in the absence of other clinical events should have the diagnostic investigations such as the cardiac magnetic resonance and serum Lyso-Gb3. They should initiate ERT as soon as possible 32.

Concerning directions for future research, migalastat (Galafold) was approved by the U.S. Food and Drug Administration as a new chaperone therapy for FD patients which could reduce Gb3 accumulation in kidney capillaries. Studies by Hughes et al. and Germain et al. showed that migalastat has a better effect on LVH improvement 33,34 than ERT treatment. It is worthy to conduct a similar meta-analysis as the one presented in our article if there are more studies conducted on the effects of migalastat in the future.

There are some limitations in our study. First, our study is a retrospective study. Because FD is a rare disease, it is difficult to find enough patients for study, and it is not possible to conduct a long-term follow-up study. Second, there are not many studies focusing on the LVMI with or without ERT. The missing data in some studies limits our sample size enrollment. The last limitation is regarding current treatment guidelines. There are more male and classical phenotype patients in ERT treatment groups as compared to treatment-naïve groups with more female and non-classic phenotype patients. The difference between these two groups increases the heterogeneity.

## Conclusion

Our meta-analysis, based on the currently available data, showed that ERT for Fabry disease has a beneficial effect on LVH. Female carriers and atypically affected males can be started on ERT as soon as a diagnosis is made. Further studies are warranted to support the role of ERT in LVH prevention.

## Figures and Tables

**Table 1:** Basic patient and studies characteristic

	ERT	Naïve	Total
Number of patients	267	285	552
Mean age (years)	40.4	46.2	43.4
Mean follow-up (years)	4.1	4.1	4.1
Gender (male # of percentage)	147 (55.1%)	127 (44.6%)	274 (49.6%)
		
	4 years after ERT	Before ERT	
Number of patients	214	228	
Mean age (years)	45.2	41.3	
Gender (male # of percentage)	100 (46.7%)	110 (48.2%)	

## Abbreviations

CI: Confidence intervals
ERT: Enzyme replacement therapy
FD: Fabry disease
IVS: Interventricular septum
LV: Left ventricular
LVH: Left ventricular hypertrophy
LVMI: Left ventricular mass index
NCBI: National Center for Biotechnology Information
RCT: Randomized controlled trials
